# FXR expression is associated with dysregulated glucose and lipid levels in the offspring kidney induced by maternal obesity

**DOI:** 10.1186/s12986-015-0032-3

**Published:** 2015-11-14

**Authors:** Sarah J. Glastras, Muh Geot Wong, Hui Chen, Jie Zhang, Amgad Zaky, Carol A. Pollock, Sonia Saad

**Affiliations:** Kolling Institute, Department of Medicine, University of Sydney, Sydney, Australia; Department of Endocrinology, Diabetes and Metabolism, Royal North Shore Hospital, St Leonards, Australia; School of Life Sciences, Faculty of Science, University of Technology Sydney, Sydney, Australia

**Keywords:** Maternal obesity, Developmental programming, Renal injury, Chronic kidney disease, Farnesoid X receptor, Nuclear hormone receptor

## Abstract

**Background:**

Maternal obesity is associated with dysregulation of glucose and lipid metabolism with consequent exposure of the fetus to an abnormal metabolic milieu. It is recognized that maternal obesity predisposes offspring to chronic kidney disease (CKD). We aimed to determine whether the nuclear Farnesoid X receptor (FXR), known to play a role in maintaining homeostasis of glucose and lipid metabolism, is involved in renal injury in offspring of obese mothers.

**Methods:**

Maternal obesity was established in a rat model by feeding dams with high-fat diet prior to and during pregnancy and lactation. The offspring’s kidneys were examined at postnatal Day 1and Day 20. Human kidney 2 (HK2) cells were exposed to high glucose with or without the FXR agonist GW4064 or when FXR mRNA was silenced.

**Results:**

Glucose intolerance in the offspring of obese mothers was evident at weaning, with associated downregulation of renal FXR expression and upregulation of monocyte chemoattractant protein-1 (MCP-1) and transforming growth factor-β1 (TGF-β1). HK2 cells exposed to high glucose had reduced FXR expression and increased MCP-1, TGF-β1, fibronectin and collagen IV expression, which was reversed in the presence of GW4064. FXR-silenced HK2 cells had amplified pro-inflammatory and pro-fibrotic markers under high glucose conditions.

**Conclusions:**

Maternal obesity influences renal expression of pro-inflammatory and fibrotic factors that predispose the offspring to CKD. This was associated with the downregulation of the renal FXR expression suggesting a potential protective role for FXR.

**Electronic supplementary material:**

The online version of this article (doi:10.1186/s12986-015-0032-3) contains supplementary material, which is available to authorized users.

## Introduction

Obesity is increasing in pandemic proportions worldwide, both in Western countries such as the United States and Australia but also in developing countries [1, 2]. Obesity clearly presents a personal risk for a range of future chronic disease, but when occurs during pregnancy and lactation has been shown to increase the risk of future chronic disease in the offspring [3–5]. Developmental programming is increasingly recognized as an important determinant of offspring health [3, 4, 6–10]. Indeed, offspring born to obese mothers are at increased risk of cardiovascular disease, stroke, diabetes and obesity itself [3, 5, 11]. There are many factors that influence disease risk, which include genetic predisposition, postnatal diet and lifestyle as well as maternal factors that affect the *in utero* environment. Animal models are a useful means by which to examine the effect of maternal obesity during pregnancy and lactation on offspring without the confounding factors of genetic background and adult lifestyle.

Nuclear hormone receptors are ligand-activated transcription factors that regulate gene expression and are involved in many developmental, metabolic and endocrine functions [12]. The Farnesoid X receptor (FXR) is a nuclear hormone receptor critical in bile acid, glucose and lipid metabolism [12–18]. FXR is found in several tissues outside the enterohepatic system and in particular, is highly expressed in the kidney, with expression detected in glomeruli, mesangial cells, podocytes and proximal tubular cells (PTCs) [19]. FXR activation is associated with improved glucose metabolism and insulin sensitivity [16, 20]. Within the kidney, FXR activation downregulates lipogenic and fibrotic genes and reduces diabetes and obesity-related changes including glomerulosclerosis, tubulointerstitial fibrosis and proteinuria [21–26]. FXR null mice are at increased risk of developing diabetes and obesity-related nephropathy [25–27]. FXR is also important in the regulation of urine volume, and its activation increases the ability of renal tubules to concentrate urine [27].

Maternal obesity is likely to affect future kidney health in offspring. Several studies have shown that offspring born to diabetic mothers are at increased risk of hypertension, hyperfiltration and chronic kidney disease (CKD) [28]. Maternal obesity is yet more complex with dysregulated glucose, lipid and hormonal changes. Despite one report of a 22 % increased risk of CKD in offspring of obese mothers, more conclusive evidence is lacking [29]. Obesity itself may lead to glomerulosclerosis and proteinuria [30]. Obesity related inflammation plays a significant role in the development and progression of renal damage in obesity [31–33]. There is also significantly more renal lipid accumulation within the kidneys of obese animals [23, 34].

In this study, we aimed to investigate the effect of maternal obesity on the risk of CKD in the offspring in a rodent model and determine whether dysregulation of FXR expression is involved in this process. We hypothesized that FXR may be an important mediator of inflammatory and fibrotic pathways known to be involved in CKD and that maternal obesity may modulate these pathways. Therefore, we exposed HK2 cells to high ambient glucose to determine the effect on FXR expression, and investigate the effect of FXR activation and silencing on fibrotic and inflammatory mediators in those cells.

## Materials and methods

### Animal model of maternal obesity

Twenty virgin outbred female Sprague Dawley rats (8-week old, Animal Resource Centre Pty Ltd, Perth, Australia) were maintained on 12 h light/dark cycle, 20 ± 2 °C. They were randomly assigned to one of 2 groups with equal average body weight: (1) standard chow (11 kJ/g, 14 % fat, Gordon’s Speciality Stockfeeds, NSW, Australia) or (2) high-fat diet (HFD, 20 kJ/g, 43 % fat; Specialty Feeds, Perth, WA) [8]. After 5 weeks, females were mated, and, housed individually. The same diet was maintained during pregnancy and lactation until weaning. The study was approved by the Animal Care and Ethics Committee of the University of Technology Sydney (ACEC# 2009–350), and the “Australian code of practice for the care and use of animals for scientific purposes” (NHM&RC, Australia) were followed. At birth, litter size was adjusted to 10 pups per mother (gender ratio: 1/1). An intraperitoneal glucose tolerance test (IPGTT) was performed at postnatal day 20. AUC was calculated for each rat. At postnatal Day 1 or Day 20, tissues from male offspring (*n* = 8) were collected. Day 1 offspring were killed by direct decapitation. Day 20 offspring were anaesthetized (Pentothal® 0.1 mg/g, i.p., Abbott Australasia, NSW, Australia) after overnight fasting. Blood were collected through cardiac puncture and blood glucose was measured immediately (Accu-Chek®, Roche Diagnostics, NJ, USA). Plasma was stored for lipids and hormonal determination. Left kidneys were snap frozen in liquid nitrogen and kept at −80 °C for mRNA and protein quantification. Right kidneys were embedded in paraffin and sectioned for immunohistochemistry staining.

### Biochemical analyses

Insulin concentrations were assessed using a radioimmunoassay kit (Linco, St Charles, USA). Plasma non-esterified fatty acid (NEFA) was measured using NEFA kit (WAKO, Osaka, Japan). In order to examine effects on renal function. Cystatin C was measured by immunoassay as per manufacturer’s instructions (R&D Systems, Minneapolis, USA).

### In vitro cell culture of HK-2 cells

HK-2 cells of a human proximal epithelial cell line from American Type Cell Collection (ATCC, USA) were used as previously described [35]. Cells were grown in 10 cm tissue culture dishes (Becton, Dickinson, NJ, USA), in keratinocyte serum-free media (KSFM) supplemented with bovine pituitary extract and epidermal growth factor (GIBCO). Initial ‘dose-response’ experiments for FXR agonist GW4064 (Sigma Aldrich, USA) were undertaken to determine the concentration at which GW4064 maximally stimulated FXR protein expression, with minimal cytotoxicity. Exposure of HK2 cells to 1 μM of GW 4064 resulted in no cell toxicity. When the HK-2 cells were 60–70 % confluent, they were exposed to the following experimental conditions for 24 h prior to RNA extraction and 48 h before protein extraction: 1) 5 mM D-glucose (ICN Biomedicals, Ohio, USA) (vehicle control); 2) 30 mM D-glucose; 3) 1 μM GW 4064; 4) 1 μM GW 4064 + 30 mM D-glucose and 5) 5 mM D-glucose + 25 mM L-glucose as an osmotic control (ICN Biomedicals, Ohio, USA).

### Gene silencing

Gene silencing was performed using small interfering RNA (siRNA) strategies. Twenty-seven-mer double-stranded RNA molecules were chemically synthesized (Shanghai GenePharma Co, Ltd, Shanghai, China). The complementary oligonucleotides were 2′-deprotected, annealed, and purified by the manufacturer. The sequence specifically targeting human FXR (NR1H4) (accession no. NM_005123) was 5′-GAUUGUUACUUCAACUCUATT-3′, 5′ UAGAAUUGAAGUAACAAUCTT 3′. HK-2 cells were plated in a 6-well plate. FXR siRNA (80 nmol/L) was introduced into HK-2 cells using Lipofectamine 2000. In parallel, cells were transfected with a non-specific siRNA which served as a control. Twenty-four hours after transfection, both the control and the FXR silenced cells were exposed to 5 mM or 30 mM D-glucose for 48 h. Silencing was confirmed by mRNA and protein expression.

### Relative quantitative real-time PCR (RT-PCR)

RNA was extracted using RNeasy mini kit (Qiagen, Valencia, CA, USA). cDNA was generated using Transcriptor First Strand cDNA Synthesis Kits (Roche Diagnostics, Mannheim, Germany). RT-PCR was performed using an ABI Prism 7900 HT Sequence Detection System (Applied Biosystems, Foster City, CA, USA). SYBR GreenER qPCR Supermix (Invitrogen) with unlabeled PCR primers was used for the animal experiments (Additional file 1, Table S1). Taqman Gene Expression Master Mix (Applied Biosystems) and gene-specific expression assays containing two unlabeled PCR primers and FAM dye-labeled Taqman MGB probes was used for *in vitro* work (Additional file 1: Table S2). Results are presented as fold change compared to control after normalization to β-Actin.

### Immunohistochemistry (IHC)

Formalin-fixed paraffin kidney sections were deparaffinised and incubated with the polyclonal primary antibody anti-FXR (1:50 dilution, Abcam Ltd, Cambridge), anti-fibronectin (dilution 1:1,000, Abcam) and anti-collagen IV (dilution 1:1,000, Abcam) overnight, followed by horseradish peroxidase anti-rabbit Envision system (Dako Cytochemistry, Tokyo, Japan) the following day. Staining was developed with 3.3diaminobenzidine tetrahydrochloride (Dako Cytochemistry, Tokyo, Japan) before counterstaining with Mayer’s haematoxylin stains. Antibody against rabbit IgG was used as a negative control. Images were analysed using an Olympus microscope (Olympus, Japan) and Image J software.

### Immunofluorescence (IF)

Formalin-fixed paraffin kidney sections were deparaffinised and epitope was retrieved by boiling the tissues in sodium citrate (pH 6.0) for 20 min. Cells were permeabilised using 0.2 % Triton-X100 (Sigma Aldrich). Tissues were then blocked in 10 % blocking solution (Dako Cytochemistry) and incubated with SREBP1 primary antibody (1 in 100 dilution over night at 4 °C) (Abcam, Cambridge, USA). The immunohistochemical expression was visualized by means of anti-rabbit Alexa® Fluor 488 (FITC alternative, green fluorescent dye, Dako Cytochemistry) at 1:2000 dilution in serum for 1 h.

### Western blot analysis

Protein was extracted from HK-2 PTCs lysate and western blotting were performed as previously described [36]. Briefly, 15 mg of total cell protein was mixed with 6X Laemmli sample buffer containing mercapto-ethanol and heated at 95 °C for 10 min. Samples were then analyzed by SDS-PAGE in 2.5 to 10 % Novex Bolt mini gels (Life Technologies, California, USA) and electroblotted to Hybond Nitrocellulose membranes (Amersham Pharmacia Biotech, Bucks, UK). Membranes were blocked in Tris-buffered saline containing 0.2 % Tween 20 (TTBS) in 5 % skim milk for 2 h. Then primary antibodies against FXR 1:1000 (Abcam); fibronectin 1:1000 (Sigma Aldrich), collagen IV 1:5000 (Abcam) and SREBP1c 1:1000 (Abcam) were added and incubated overnight at 4 °C. Membranes were washed and incubated with horseradish peroxidase-conjugated secondary antibody for 2 h at room temperature, and washed. The membranes then were reprobed with β-Actin 1:10000 (Santa Cruz, CA). Protein bands were visualized using the enhanced chemiluminescence (ECL) detection system (Amersham Pharmacia Biotech). The bands corresponding to fibronectin (220 kDa), collagen IV (250 kDa), FXR (69 kDa), SREBP1c (120 kDa) and β-Actin (42 kDa) were captured using LAS 4000 (Fujifilm, Tokyo, Japan), corrected for β-actin as a loading control. They were then analyzed using ImageJ software (v1.26r, National Institutes of Health, USA).

### Statistical analysis

Results are expressed as mean ± SEM. The differences between the two groups were analysed by independent Student’s t-test. When there were more than 2 groups, one-way analysis of variance (ANOVA) was used, with post-hoc Fisher’s protected least-significant difference test (Prism 5, GraphPad Software, USA). *P* < 0.05 was considered significant.

## Results

### Effect of maternal obesity on the offspring of obese mothers

Offspring of obese mothers had no difference in body weight at postnatal Day 1, compared to offspring of lean mothers (6.65 ± 0.42 vs. 6.68 ± 0.57 g). Similarly, no differences in blood glucose or insulin levels were found (blood glucose: 5.15 ± 0.58 vs. 4.47 ± 0.49 mmol/L, plasma insulin 0.21 ± 0.05 vs. 0.18 ± 0.04 ng/ml). However at postnatal Day 20, offspring of the obese mothers had significantly greater body weight compared to the offspring of lean mothers (*p* < 0.01; Table 1). In addition, offspring of obese mothers had increased adiposity; there was a 3-fold increase in retroperitoneal fat (*p* < 0.01) and a 4-fold increase in epididymal white adipose tissue (*p* < 0.01). The liver and kidneys were also heavier in offspring of the obese mothers (*p* < 0.05).

**Table 1:** Weight measurements and blood test parameters at postnatal day 20

	Control	Obese
Body weight (g)	59.3 ± 0.5	71.5 ± 2.8**
Retroperitoneal fat (mg)	71.5 ± 3.8	287.9 ± 24.6**
Epididymal fat (mg)	95.5 ± 4.9	483.4 ± 45.5**
Liver (g)	2.80 ± 0.04	3.48 ± 0.17*
Kidney (mg)	347.3 ± 5.9	375.3 ± 11.4*
Fasting blood glucose (mM)	5.54 ± 0.16	6.11 ± 0.09**
Plasma insulin (ng/ml)	0.37 ± 0.07	0.60 ± 0.07*
Plasma triglycerides (mM)	0.21 ± 0.03	1.29 ± 0.24**
Plasma fatty acids (mM)	0.94 ± 0.05	1.41 ± 0.14**

The data were analyzed by Student’s t-test *p < 0.05, **p < 0.01, *N* = 8–10 per group

After overnight fasting, blood glucose levels were higher in offspring of obese mothers (*p* < 0.01, Table 1). Likewise, plasma triglycerides and NEFA were increased significantly in obese mothers’ offspring at Day 20 (*p* < 0.01).

**Fig. 1:**
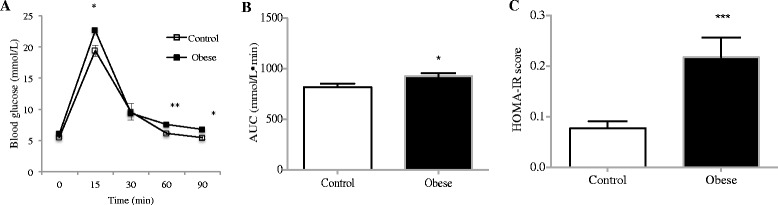
Offspring of obese mothers have reduced glucose tolerance at weaning. IPGTT at postnatal Day 20 (**a**). AUC depicted in (**b**). **c** Insulin resistance measured by HOMA-IR. *N* = 8 per group. *p < 0.05, **p < 0.01, ***p < 0.001

At Day 20, offspring of obese mothers had significantly higher blood glucose concentrations at 15, 30, and 90 min during the IPGTT (*p* < 0.01, *p* < 0.05, *p* < 0.05, respectively, Fig. 1a). The AUC value was significantly higher in offspring of obese mothers (*p* < 0.05, Fig. 1b), suggesting impaired glucose metabolism in offspring of obese mothers. Insulin resistance, as measured by HOMA-IR was elevated in offspring of obese mothers (*p* < 0.001, Fig. 1c). Renal function, as measured by serum Cystatin C was not altered by maternal obesity (see Additional file 1: Figure S1).

### FXR and SREBP1c expression in the kidneys at postnatal Day 1 and Day 20

**Fig. 2:**
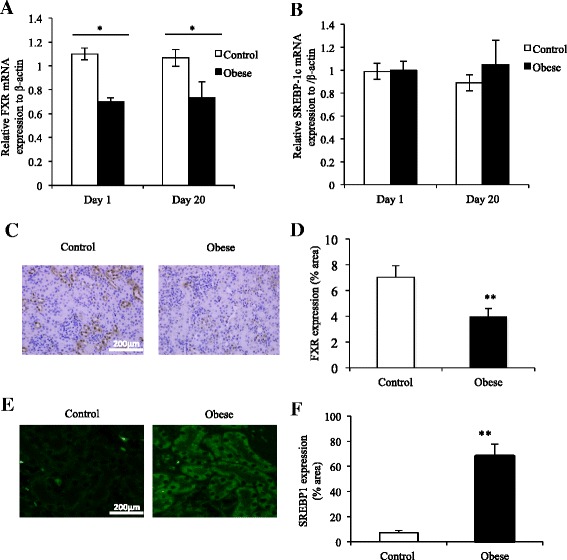
FXR was downregulated in the kidneys of offspring of obese mothers at postnatal Days 1 and 20. mRNA expression of FXR (**a**) and its downstream target SREBP-1c (**b**) at postnatal Days 1 and 20 (**b**). IHC staining (**c** and **e**) and quantification (**d** and **f**) of FXR and SREBP1c at Day 20. Images shown at 400x magnification. *N* = 5–7 per group, *p < 0.05 and **P < 0.01

Renal expression of FXR mRNA was reduced in offspring of obese mothers compared to offspring of lean mothers at Day 1 (*p* < 0.05, Fig. 2a). This effect was sustained to Day 20 (*p* < 0.05, Fig. 2a). Renal FXR protein change was consistent with mRNA changes at Day 20 (*p* < 0.01, Fig. 2c and d). FXR activation is known to inhibit sterol regulatory element binding protein-1c (SREBP-1c) expression [37]. Although renal SREBP1c mRNA levels were not changed in offspring of the obese mothers at Day 1 or Day 20, the levels of SREBP1c protein expression were upregulated in the kidney from the offspring of the obese mothers at Day 20 as expected (*p* < 0.01, Fig. 2b, e and f).

### Offspring born to obese mothers had increased renal MCP-1 and TGF-β1 mRNA levels

**Fig. 3:**
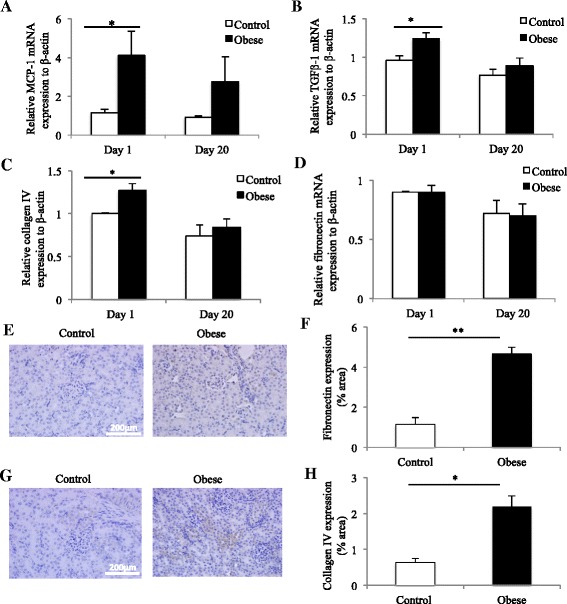
RNA expression of MCP-1 and TGF-β was upregulated in the kidneys of offspring of obese mothers at Day 1 but not at Day 20. Collagen IV but not fibronectin mRNA was increased in offspring of obese mothers at Day 1 but not at Day 20. However, cortical protein expression of fibronectin and Collagen IV was increased in offspring of obese mothers at Day 20. Renal mRNA expression of MCP-1 (**a**), TGF-β1 (**b**), Collagen IV (**c**) and fibronectin (**d**) at postnatal Day 1 and Day 20. Protein expression of fibronectin (**e**) and Collagen IV (**g**) in offspring of obese mothers at Day 20. Graphical quantitation of fibronectin and Collagen IV staining are shown in (**f**) and (**h**) respectively. *N* = 5–7 per group, *p <0.05, *p <0.01

Renal monocyte chemoattractant protein-1 (MCP-1) was increased by four times in the offspring of the obese mothers compared to the control at Day 1 (*p* < 0.05; Fig. 3a). TGF-β1 was also increased in the offspring of obese mothers at Day 1 (*p* < 0.05; Fig. 3b). There was no statistically significance in MCP-1 and TGF-β1 expression at Day 20,

### Renal markers of extracellular matrix accumulation in offspring

There was no change in in fibronectin mRNA expression at either Day 1 or Day 20 in offspring of obese versus lean mothers (Fig. 3c). Interestingly, there was an increase in collagen IV protein levels at Day 20 as measured by immunohistochemistry staining (*p* < 0.01; Fig. 3e and f). There was an increase in collagen IV mRNA expression at Day 1 but no difference at Day 20 (Fig. 3d). At the protein level, there was an increase in fibronectin levels at Day 20 (Fig. 3g and h).

### High glucose down-regulates FXR expression and regulates its target genes in human HK2 cells

**Fig. 4:**
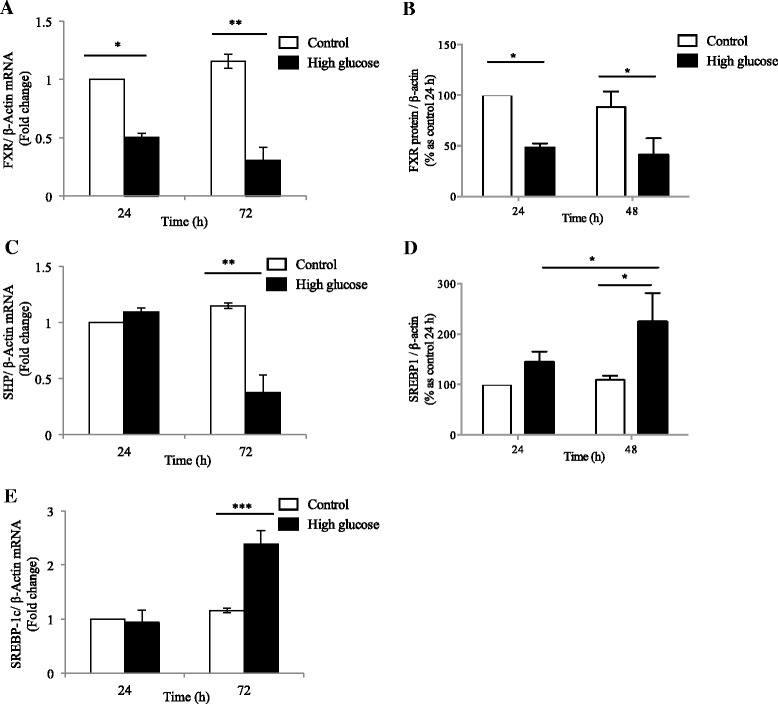
Effect of high glucose on FXR expression in HK-2 cells. HK-2 cells were exposed to 5 mM D-glucose (*white open bar*) or 30 mM D-glucose (*black solid bar*) for 24 or 72 h. mRNAexpression of FXR (**a**), SREBP-1c (**c**) and SHP (**e**) is shown at 24 and 72 h. Protein expression of FXR (**b**) and SREBP1 (**d**) is also shown at 24 and 48 h. *N* = 3 per group, *p <0.05, **p <0.01, ***p <0.001

Exposure to high glucose (30 mM D-glucose) significantly suppressed FXR mRNA expression by half and 2/3 at 24 and 72 h respectively (*p* < 0.05, *p* < 0.01 respectively, Fig. 4a). This effect was not observed in cells cultured in 25 mM L-glucose + 5 mM D-glucose, which served as an osmotic control. The same effect was seen at the protein level by Western blot, whereby FXR was reduced in culture exposed to high glucose at both 24 and 48 h (*p* < 0.05, Fig. 4b). Given the known reciprocal relationship between FXR and SREBP-1c and small heterodimer protein (SHP) activation, SREBP-1c and SHP were examined. At 72 h, SREBP-1c mRNA was markedly upregulated in the HK-2 cells exposed to high glucose compared to those exposed to 5 mM D-glucose (*p* < 0.001; Fig. 4c). SREBP-1c protein was significantly increased at 48 h when exposed to high glucose compared to control (*p* < 0.05, Fig. 4d). No difference in SHP mRNA expression in low or high glucose at 24 h was seen (Fig. 4b). However, 72-hours exposure to high glucose was associated with down-regulation of SHP mRNA compared to cells exposed to 5 mM D-glucose (*p* < 0.01; Fig. 4e).

### FXR agonist inhibits high glucose-induced fibronectin and collagen IV expression

**Fig. 5:**
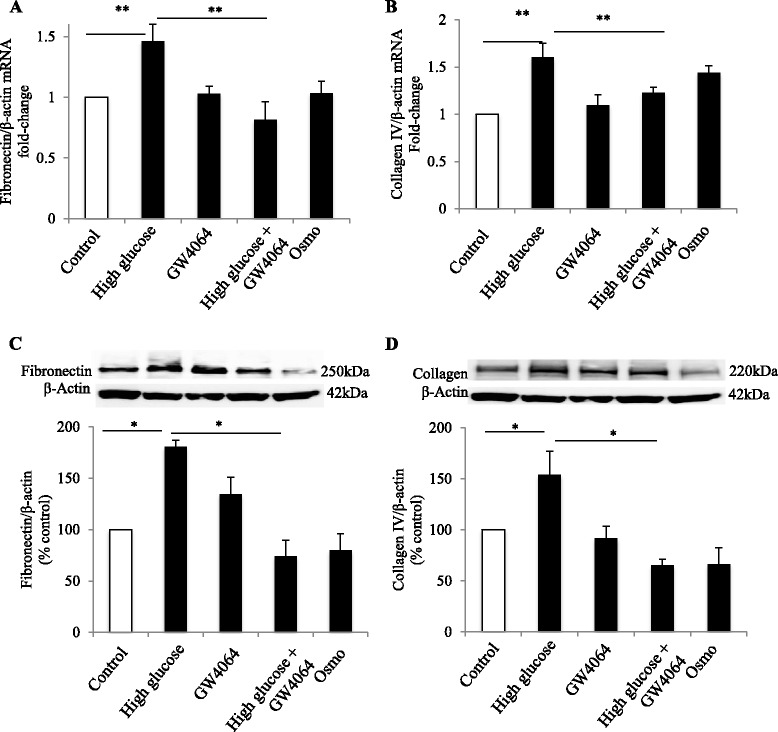
Suppression of high glucose-induced fibronectin and collagen-IV expression by GW4064 in HK-2 cells. mRNA and protein expression of fibronectin (**a** and **c**) and Type IV collagen (**b** and **d**) exposed to high-glucose with and without the presence of GW4064 in HK-2 cells. *N* = 4 per group, *p < 0.05; **p < 0.01

The extracellular matrix proteins fibronectin and collagen IV are commonly overexpressed in the early stages of CKD. To determine the effect of high glucose on the expression of fibronectin and collagen mRNA, HK-2 cells were exposed to high glucose for 24 h. This resulted in an increase in fibronectin and collagen IV compared to the vehicle control (*p* < 0.05, Fig. 5a and b respectively). Co-incubation with GW4064 reversed the effect of high glucose on mRNA expression of fibronectin and collagen IV (*p* < 0.01, Fig. 5a and b). Similarly, protein expression of both fibronectin and collagen IV was reduced (*p* < 0.05, Fig. 5c and d).

To confirm this finding, protein expression of fibronectin and collagen IV was examined in HK-2 cells after 48 h incubation with high glucose in the presence or absence of GW4064. Fibronectin protein expression was nearly doubled compared to the control cells when exposed to high glucose (*p* < 0.01, Fig. 5c). Similarly, type IV collagen production was also significantly increased in the HK-2 cells by high glucose exposure (*p* < 0.05, Fig. 5d). GW4064 suppressed high glucose-induced fibronectin and type IV collagen production in the HK-2 cells (*p* < 0.01, Fig. 5c and *p* < 0.05, Fig. 5d respectively).

### FXR agonist inhibits high glucose-induced TGF-β1 and MCP-1 expression

**Fig. 6:**
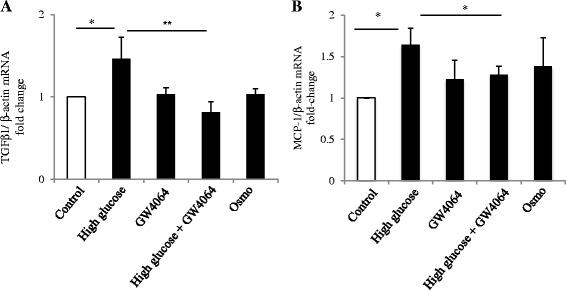
Exposure of HK-2 cells to high glucose increases TGF- β and MCP-1 mRNA expression and GW4064 ameliorates this effect. mRNA expression of TGF-β (**a**) and MCP-1 (**b**) in HK-2 cells exposed to high glucose with and without the presence of GW4064. *N* = 4–5 per group, *p < 0.05, **p < 0.01

TGF-β1 mRNA expression was significantly increased following 24 h exposure to high glucose compared to the control group (p <0.05; Fig. 6a), which was inhibited by GW4064 (*p* < 0.01). MCP-1 mRNA expression was also significantly increased by high glucose, but not ameliorated by additional GW4064 co-incubation (Fig. 6b).

### FXR silenced cells had increased fibronectin, type IV collagen and SREBP-1 expression

FXR silencing was confirmed by both mRNA and protein expression of FXR. A 50–60 % reduction of FXR protein expression was achieved in all experiments compared to the control (data not shown).

**Fig. 7:**
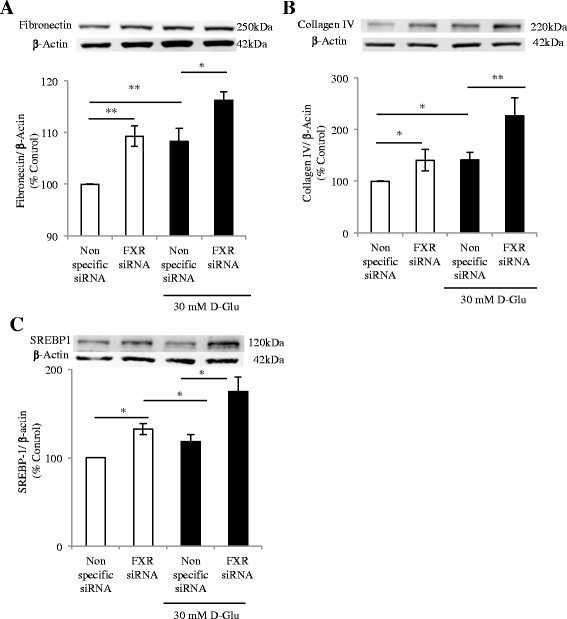
FXR silenced cells exhibit increased ECM and SREBP-1 expression, further upregulated by exposure to high glucose conditions. Protein levels of fibronectin (**a**), type IV collagen (**b**), and SREBP-1c (**c**) in FXR silenced cells exposed to 5 mM D-glucose (*black solid bar*) and 30 mM D-glucose (*white empty bar*) for 72 h. *N* = 4–5 per group, *p < 0.05, **p < 0.01

There was increased extracellular matrix markers in FXR silenced HK-2 cells compared to cells transfected with non-specific siRNA. Both fibronectin and type IV collagen expression were increased in FXR silenced cells (*p* < 0.01, *p* < 0.05, Fig. 7a and b, respectively). Exposure to high glucose conditions (30 mM D-glucose for 72 h) increased fibronectin expression in both normal and FXR-silenced cells but significantly more so in the FXR-silenced cells (*p* < 0.05, FXR-silenced vs. normal). Similarly, type IV collagen expression was increased in HK-2 when FXR was silenced (*p* < 0.05) or exposed to high glucose (*p* < 0.05), which was further increased to in FXR silenced cells exposed to high glucose (*p* < 0.05, Fig. 7b).

Increased SREBP-1 expression was found in FXR silenced cells compared to cells treated with non-specific siRNA (*p* < 0.05, Fig. 7c). Exposure to 30 mM D-glucose further increased SREBP-1 expression in cells with silenced FXR, confirming that FXR can inhibit the expression of SREBP-1 (Fig. 7c).

## Discussion

This study demonstrates that maternal obesity has significant adverse effects on the offspring’s kidneys and may increase the risk of kidney damage later in life. This study suggests that suppressed renal FXR is critical for maternal obesity-induced nephropathy in offspring, which has not been reported previously.

Growing evidence has suggested that dysregulation in glucose as well as lipid metabolism may play a major role in the pathogenesis of obesity-related nephropathy [23, 25, 26, 34, 38–41]. Despite the known relationship between obesity-related nephropathy and glucose dysregulation and dyslipidemia, the contribution of FXR regulation to renal damage has not been well characterized. Our *in vivo* study showed that offspring of obese mothers have reduced renal expression of FXR until at least weaning in rats. FXR is mainly expressed in the tubules, but not in the glomeruli, as shown in this study and reported previously by others [42]. *In vitro* study using human proximal tubule epithelial cell line demonstrated that high ambient glucose inhibits FXR expression and that the activation of FXR mitigates the effects of high ambient glucose to increase pro-inflammatory, pro-fibrotic mediators and extracellular matrix accumulation of collagen IV and fibronectin, which are the first step towards the development of CKD. Furthermore, FXR silencing markedly enhanced the effect of high glucose on inflammatory, fibrotic and lipid pathways in PTCs. These results suggest a significant role of FXR in modulating inflammation and fibrosis within PTCs under high glucose conditions. This is not surprising given FXR has already been shown to play a role in the development of hepatic fibrosis [20, 21, 43].

Bile acids can cross the blood-placenta barrier. In addition, fetal and maternal bile acid levels are well correlated. Bile acid accumulation in the bloodstream induces intrahepatic cholestasis of pregnancy (ICP). A recent study by Papacleovoulou et al. showed that children born to pregnant women with ICP had higher body mass indices and increased fasting hyperinsulinaemia at age 16 despite normal birth weight [44]. The offspring of mothers with ICP were more prone to obesity and diabetes [44]. In our study, offspring were exposed to overnutrition due to maternal obesity including increased fatty acids, cholesterol, glucose and other nutrients, all of which may impact upon FXR expression in the kidney.

The role of inflammation in the development of CKD is increasingly appreciated. We recently reported that inflammatory mediators are important for the development of fibrosis within PTCs [45]. We showed that in the presence of high glucose, transforming growth factor-β1 (TGF-β1) expression is induced and in particular, TGF-β1 plays a major role in upregulating other proinflammatory cytokines, including MCP-1 [46]. These cytokines directly regulate the activity of pro-fibrotic markers, including fibronectin and collagen IV [46]. FXR has been shown to negatively interfere with the inflammatory response [47]. Wang et al. showed that obese mice had increased expression of MCP-1 and reduced expression of anti-inflammatory cytokine Kruppel-like factor 4 within their kidneys [23]. In this study, offspring of obese mothers had increased MCP-1 and TGF-β1 despite no difference in their body weight at postnatal Day 1 suggesting an intrauterine programming. Our *in vitro* model demonstrated that activation of FXR using agonist can reduce expression of MCP-1 and TGF-β1 as well as fibrotic markers fibronectin and collagen IV; while silencing FXR had the opposite effect. Similarly, the FXR ligand (6-ethyl chenodeoxycholic acid) has been shown to have anti-fibrotic, anti-inflammatory and anti-apoptotic effects in cisplatin-induced HK-2 cells [48]. Interestingly, here in rats, despite *in utero* exposure to high-fat diet there appeared to be minimal effects on pro-fibrotic markers. It is likely that fibrotic changes would be evident at later time points as it was demonstrated in the study performed by Hao et al. showing significant extracellular matrix (ECM) changes after 3 months post-birth [48].

Previous studies have suggested that FXR activation has beneficial effects on the kidney by altering the expression of SREBP-1 and its lipogenic target genes [50]. SREBP-1 is a known key transcriptional factor linking fatty acid and lipid accumulation to ECM deposition [15, 40]. In SREBP-1 transgenic mice, overexpression of SREBP-1 in the kidney caused lipid accumulation and increased expression of profibrotic cytokines, which induced ECM accumulation, mesangial expansion, glomerulosclerosis and proteinuria [41]. SREBP-1c lies downstream of FXR [37]. SREBP-1c is known to be expressed in human and animal tissues, in particular kidneys [51, 52]. Although mRNA expression of FXR in mouse PTCs is five times more than in glomeruli, there is a lack of evidence linking FXR with SREBP-1c in kidney PTCs [22]. In our HK2 PTCs, high glucose was associated with downregulation of FXR and SHP with upregulation of SREBP-1c. Similarly, the downregulation of FXR was associated with an upregulation of SREBP1c protein expression in offspring from obese rats at weaning.

Suckling from obese mothers may confound the maternal effects incurred *in utero*. Previous studies addressed this confounding problem by cross-fostering techniques [4], thereafter to conclude that i*n utero* exposure has independent effects on organ development and metabolism. Interestingly changes in FXR were demonstrated at Day 1 before significant neonatal milk consumption. In addition, changes in MCP-1, TGF-β1 and collagen IV were also evident at Day 1. These changes had mostly normalized by postnatal Day 20, suggesting postnatal milk intake may have ameliorated the intrauterine effect due to maternal HFD consumption. It is well documented that breastfeeding has significant protective effects against obesity and its related metabolic effects [53–55] possibly due to increased amounts of antioxidants in the breast milk [56]. Nevertheless, we postulate that the future development of CKD may require a second insult such as HFD consumption. Early metabolic changes and changes in renal inflammatory and fibrotic markers at birth in the absence of overt renal functional changes may suggest increased risk of future CKD in adulthood.

Offspring of obese mothers had glucose intolerance at weaning; therefore we pursued the role of FXR in glucose-mediated renal damage in our *in vitro* model. Indeed, we showed that FXR is linked to glucose-mediated inflammatory and fibrotic changes in PTCs. Taken together, basal FXR expression is important in maintaining ECM homeostasis and high glucose suppresses FXR expression. In addition, an FXR agonist can abrogate high glucose-induced ECM production. However, the offspring of obese mothers also had impaired lipid metabolism at Day 20. We did not investigate the role of FXR in lipid/cholesterol-mediated damage to PTCs here. Other nuclear hormone receptors such as vitamin D receptor (VDR) and peroxisome-proliferator-associated receptors (PPARs) are known to play a role in renal inflammation, oxidative stress, fibrosis and renal lipid deposition and their activation may mitigate nephropathy in diabetic animals [57]. Nuclear receptor activation of FXR, VDR and PPAR tend to have common pathways leading to modulation of glucose metabolism, lipid metabolism and immune response [57–59].

FXR is a potential pharmacological target for the treatment of obesity and metabolic syndrome due to its regulation of bile acids, lipids and glucose metabolism [15, 17, 18, 20, 23]. Weight reduction induced by sleeve gastrectomy was recently demonstrated to be critically dependent upon FXR activation [60]. Indeed, FXR knockout mice with dietary obesity failed to lose significant weight with sleeve gastrectomy compared to their wild-type counterparts. The therapeutic benefit of FXR agonists is only just being explored. A clinical trial investigating the effect of the FXR agonist INT-747 in patients with type 2 diabetes and non-alcoholic fatty liver disease, concluded that FXR activation improved insulin sensitivity and reduced markers of liver inflammation and fibrosis [61]. More clinical trials are underway to determine the effect of FXR agonist in the setting of diabetes and non-alcoholic fatty liver disease [62].

Several studies have shown that FXR activation can ameliorate diabetic nephropathy by modulating lipid metabolism, fibrosis, inflammation, and oxidative stress in animals and in glomerular and mesangial cells *in vitro* [22, 63, 64]. This study suggests that this process also occurs in PTCs. Furthermore, maternal obesity can change renal FXR expression associated with dysregulated glucose and lipid levels in the offspring. Our study is the first to report the effect of maternal obesity, which is a global health issue in humans, on the offspring’s kidney, and linked it to FXR as a critical regulator of the developmental programming effect of maternal obesity on kidney health. The long term effects of a reduction of FXR level in early post-natal life on the development of CKD requires further investigation. If proven, the relative benefits of modifying FXR in the offspring of obese mothers will necessitate future study

In summary, maternal obesity downregulates renal FXR expression in the offspring kidney, which is associated with increased inflammation and early fibrotic change in the kidney at birth. Given the role that FXR plays in regulating lipid and glucose metabolism, with downstream activation of inflammatory and fibrotic pathways, the potential exists for FXR modulation to prevent and ameliorate kidney damage associated with maternal obesity.

## Additional file

Additional file 1:
**Supplementary data.** (DOCX 96 kb)
