# Supplementary material for: FXR expression is associated with dysregulated glucose and lipid levels in the offspring kidney induced by maternal obesity
Source: Nutr Metab (Lond). 2015 Nov 14;12:40. doi: 10.1186/s12986-015-0032-3 (PMC4650952; doi:10.1186/s12986-015-0032-3)
Supplement: Additional file 1: — Supplementary data. (DOCX 96 kb) [file 12986_2015_32_MOESM1_ESM.docx]

**Additional file 1: Supplementary Data**

**Table S1. Real time qPCR rat primers**

| Gene | Forward primer (5’-3’) | Reverse primer (5’-3’) |
| --- | --- | --- |
| FXR | TGACAAAGAAGCCGCGAAT | TGTAATGGTACCCAGAGGCCC |
| SREBP1c | CATGGATTGCACATTTGAAGAC | GCAGGAGAAGAGAAGCTCTCAG |
| MCP-1 | GTTGTTCACAGTTGCTGCCT | CTCTGTCATACTGGTCACTTCTAC |
| TGF-β1 | AGGACCTGGGTTGGAAGTGG | AGTTGGCATGGTAGCCCTTG |
| Collagen IV | CCATGGTCAGGACTTGGGTA | AAGGGCATGGTGCTGAACT |
| Fibronectin | CAGCCCCTGATTGGAGTC | TGGGTGACACCTGAGTGAAC |
| β-actin | GTGGGGCGCCCCAGGCACCA | CTCCTTAATGTCACGCACGATTTC |

Primer sequence for rat Farnesoid X receptor (FXR), sterol receptor element binding protein-1c (SREBP1c), monocyte chemoattractant protein (MCP)-1, transforming growth factor (TGF- **β**1), collagen IV, fibronectin, and **β**-actin genes are shown

**Table S2. Real time qPCR human primers**

| Human primers | Assay 1D | Amplicon length |
| --- | --- | --- |
| FXR (NR1H4) | Hs00231968_m1 | 85 |
| SREBP1c | Hs01088691_m1 | 90 |
| SHP (N20B2) | Hs00222677_m1 | 87 |
| Collagen IV (COL13A) | Hs00193225_m1 | 66 |
| Fibronectin | Hs01549976_m1 | 81 |
| MCP-1 | Hs00234140_m1 | 101 |
| TGF-β1 | Hs00998133_m1 | 57 |
| Human β-actin | 4333762F | 171 |

Primer assay ID and amplicon length for each human primer is listed for Farnesoid X receptor (FXR), sterol receptor element binding protein-1c (SREBP1c), small heterodimer protein (SHP), collagen IV, fibronectin, monocyte chemoattractant protein (MCP)-1, transforming growth factor (TGF- **β**1) and **β**-actin genes

**Fig. S1 Serum Cystatin C in offspring of obese and lean mothers**

Cystatin C was measured as a marker of renal function in offspring serum from control and obese mothers (N = 4-5 per group).
